# Severe Asthma Exacerbations: From Risk Factors to Precision Management Strategies

**DOI:** 10.3390/jcm15020857

**Published:** 2026-01-21

**Authors:** Marina Paredes, Jeisson Osorio, Alberto García de la Fuente, Elena Rodríguez, César Picado, Iñigo Ojanguren, Ebymar Arismendi

**Affiliations:** 1 Pulmonology Department, Hospital Clínic de Barcelona and Universitat de Barcelona, 08036 Barcelona, Spain; mparedesl@clinic.cat (M.P.); osorio@clinic.cat (J.O.);; 2 Centro de Investigación Biomédica en Red de Enfermedades Respiratorias (CIBERES), Instituto de Salud Carlos III de Madrid, 28222 Madrid, Spain; 3 Pulmonology Department, Hospital Universitari Vall d’Hebron, 08035 Barcelona, Spain

**Keywords:** exacerbation-prone, eosinophilic asthma, biomarkers, treatment, intensive care

## Abstract

**Background**: Severe asthma exacerbations (SAEs) significantly contribute to asthma-related morbidity, mortality, and healthcare burden. Despite therapeutic advances, a subset of patients remains exacerbation-prone. This review aims to summarize current evidence on risk factors, phenotypes, and biomarkers associated with SAEs, and explore personalized strategies for their acute management. **Methods**: We conducted a comprehensive literature review focusing on clinical, inflammatory, and environmental drivers of SAE. Special attention was given to Type 2 (T2) biomarkers—blood eosinophil count (BEC) and fractional exhaled nitric oxide (FeNO)—as tools for phenotyping and treatment guidance. Emerging evidence on the use of biologics during exacerbations was also analyzed. **Results**: SAEs are heterogeneous in etiology and inflammatory profile. Respiratory infections, allergen exposure, obesity, and comorbidities increase exacerbation risk. T2-high SAEs respond well to corticosteroids and biologics, whereas T2-low SAEs show limited treatment benefit. BEC and FeNO reliably predict exacerbation risk and corticosteroid responsiveness. Recent case reports suggest potential roles for anti-IL-5 and anti-thymic stromal lymphopoietin (TSLP) biologics in acute care. **Conclusions**: Biomarker-guided management of SAEs may enhance therapeutic precision and avoid overtreatment. Integrating phenotypic (observable characteristics) and endotypic (biological markers) assessment into acute care could improve patient outcomes and optimize resource use. Prospective trials are needed to confirm these approaches.

## 1. Introduction

Asthma exacerbations (AEs), particularly severe episodes [1], represent a significant concern on global health, contributing to increased morbidity, mortality, and healthcare costs, as well as low quality of life for those affected [2,3]. According to the Global Initiative for Asthma (GINA) [4], approximately 8–13% of patients experience at least one exacerbation annually. However, this prevalence may vary across different regions and populations due to epidemiological and environmental factors [5]. AEs place a considerable burden on individuals, healthcare systems, and the environment.

Severe asthma exacerbations (SAEs) significantly increase mortality, with asthma causing about 1000 deaths worldwide each day [6]. A history of prior exacerbations, particularly two or more in the previous year, is a strong predictor of future exacerbation risk [7].

SAEs involve sudden or gradual (slow) worsening of symptoms like dyspnea, wheezing, and chest tightness, often requiring systemic corticosteroids, hospitalization, or intensive care [8,9]. Despite therapeutic progress, current acute management still relies on a uniform escalation of bronchodilators and systemic corticosteroids, without considering the marked biological heterogeneity of these events. This gap limits precision in treatment decisions and disproportionately affects those with frequent and difficult-to-control exacerbations, who are typically classified as having exacerbation-prone asthma (EPA) [10], which continues to present a significant clinical challenge. EPA is generally identified by the requirement for two or more courses of systemic corticosteroids annually, even when patients are on at least two maintenance treatments, including a medium- or high-dose inhaled corticosteroid (ICS) [11,12]. Researchers have described the features of these episodes to help identify patients who may be at higher risk [13,14,15,16].

We still lack simple and reliable tools to identify different exacerbation phenotypes during acute care. Biomarkers such as blood eosinophils and FeNO are promising because they provide objective information on the underlying inflammation. However, the current evidence is still limited, and their usefulness in SAEs has not been confirmed in robust trials. For this reason, clinical guidelines have not changed the standard management of SAEs, and biomarker-guided treatment is not yet recommended.

This review seeks to synthesize the current body of literature on SAEs, with particular emphasis on risk factors, phenotypic characteristics, and the identification of existing and potential biomarkers.

## 2. Materials and Methods

A non-systematic narrative review was conducted by searching the PubMed, Embase, and Scopus databases for articles published until June 2025. The primary search terms included combinations of “asthma exacerbation”, “severe”, “risk factors”, and “exacerbation-prone asthma”. Additional articles were identified by manually reviewing reference lists of relevant publications. English- and Spanish-language studies involving adult populations and addressing clinical, immunological, or therapeutic aspects of severe asthma exacerbations were included. Priority was given to recent reviews, randomized trials, and cohort studies. No formal quality assessment or meta-analysis was performed, as this review aimed to provide a comprehensive synthesis of current evidence, emerging concepts, and expert perspectives not captured by strict systematic criteria.

## 3. Relevant Sections

### 3.1. Clinical Subtype Classification

SAEs have a significant heterogeneity in their clinical presentation and underlying pathophysiological mechanisms. In recent years, there has been increasing interest in characterizing AEs to clarify their underlying mechanisms. Phenotyping—classifying exacerbations based on observable clinical features—has become an essential element of personalized asthma management [17].

A few decades ago, some researchers proposed classifying exacerbations according to time of onset, distinguishing sudden-onset (rapidly progressing) from slow-onset (subacute) episodes. In all reported cases, the latter accounts for the majority (80%). These subgroups exhibit different clinical trajectories [18]. Sudden-onset exacerbations usually respond rapidly to initial treatment. As a result, they require lower cumulative doses of systemic corticosteroids and are associated with shorter hospital stays [19]. Regarding triggers, this smaller subgroup is more frequently associated with allergens, exercise, and psychosocial stress [20], intake of nonsteroidal anti-inflammatory drugs [21], and menstruation [22], while infections are less common. The fact that the time of onset may suggest the etiology of the exacerbation is the reason why some authors advocate for the usefulness of this classification in determining the most appropriate treatment and management approach [23,24].

Sudden-onset AEs can occur as epidemic asthma outbreaks. Epidemic asthma refers to sharp increases in asthma attacks in the population, often associated with environmental factors such as airborne pollen during thunderstorms (epidemic thunderstorm asthma) [25] or specific industrial conditions, like the documented soybean dust episodes in Barcelona, where unloading activities correlated with a rise in asthma cases [26].

### 3.2. Risk Factors

**Figure 1:**
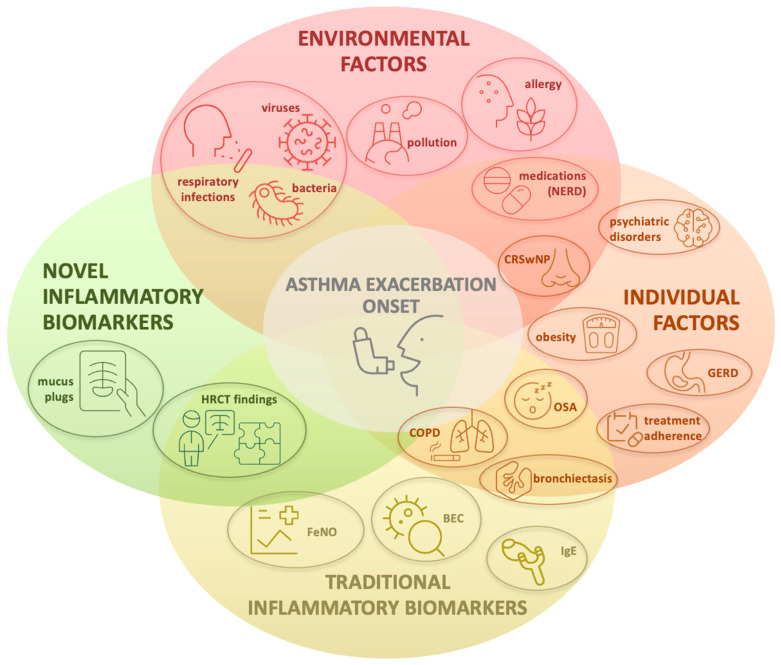
Mechanisms of asthma exacerbation’s onset. NERD: nonsteroidal anti-inflammatory drug-exacerbated respiratory disease; CRSwNP: chronic rhinosinusitis with nasal polyps; OSA: obstructive sleep apnea; GERD: gastroesophageal reflux disease; COPD: chronic obstructive pulmonary disease; FeNO: fractional exhaled nitric oxide; BEC: blood eosinophil count; IgE: immunoglobulin E; HRCT: high-resolution computed tomography.

The onset of SAEs is driven by a combination of environmental factors and patient-specific characteristics, resulting in inadequately controlled airway inflammation and subsequent clinical deterioration, as shown in Figure 1 [27,28,29,30].

#### 3.2.1. Environmental Factors

##### Respiratory Infections

Respiratory infections, particularly those of viral origin, are well-established triggers for SAEs [31]. Under normal conditions, the antiviral immune response activates the Th1 pathway, leading to the release of interferons (IFN-γ, IFN-β, and IFN-λ). These interferons limit viral entry and replication by inducing an early epithelial antiviral state. In asthmatic patients with a predominant T2 inflammatory response, experimental studies show suppression of Th1 cytokines and IL-10, resulting in reduced interferon production. This impaired antiviral response renders the airway epithelium more permissive to viral replication and favors more severe exacerbations. The impaired interferon response is accompanied by an upregulation of T2 cytokines, contributing to more severe exacerbations [32]. Additionally, the Th2 pathway drives IL-8 production, fostering neutrophil recruitment and heightened bronchial hyperresponsiveness [33]. Through the action of elastase, neutrophils release double-stranded DNA (dsDNA), forming neutrophil extracellular traps (NETs) that stimulate mucus hypersecretion and contribute to airway obstruction and mucus plug formation [34]. Although this phenomenon has traditionally been associated with T2-high asthma, recent studies indicate that the Th1-mediated antiviral response is also dysregulated in T2-low asthma [35].

When modern RT-PCR techniques are used, viruses are detected in approximately 80% of wheezing episodes in school-aged children and in about one half to three quarters of acute wheezing episodes in adults [36]. Rhinovirus is a common pathogen year-round, whereas respiratory syncytial virus, influenza, and metapneumovirus mainly peak in winter [37]. Rapid immune responses control viral replication and reduce inflammation, while slower responses can worsen symptoms. In children aged 2–17 years, although other pathogens—including respiratory syncytial virus, influenza, parainfluenza, coronavirus, adenovirus, human metapneumovirus, and bocavirus—are detectable during exacerbations, a large epidemiological study showed that only rhinoviruses remained significantly associated with asthma attacks [36]. Better understanding of respiratory virus interactions with asthma will support targeted strategies for managing virus-induced asthma exacerbations [38].

Bacterial infections can trigger SAEs through complex and multifactorial mechanisms. Atypical pathogens such as *Chlamydophila pneumoniae* and *Mycoplasma pneumoniae* have been implicated in both asthma onset and exacerbation, although evidence across studies remains inconsistent, likely due to diagnostic limitations, varying infection dynamics, and chronic colonization patterns, particularly of *C. pneumoniae* [39,40].

Typical respiratory bacteria such as *Haemophilus influenzae*, *Streptococcus pneumoniae*, and *Moraxella catarrhalis* are frequently detected, especially with polymerase chain reaction (PCR)-based methods, during SAEs and may contribute directly or in synergy with viral pathogens. For instance, co-detection of rhinovirus and *S. pneumoniae* significantly increases exacerbation risk in children compared to viral infection alone. Moreover, emerging microbiome studies indicate that specific airway bacterial profiles, such as *Moraxella*-dominant communities, are linked to increased eosinophilic activity and exacerbation susceptibility. Overall, bacterial pathogens may exacerbate asthma through direct airway inflammation, modulation of host immune responses, and amplification of viral-induced pathology [24,41].

In this context, macrolide trials provide additional insight into these mechanisms. In AMAZES, azithromycin reduced exacerbation frequency despite showing no consistent decrease in sputum bacterial load or inflammatory cell counts, suggesting that its therapeutic effects are not solely attributable to antibacterial action [39]. Earlier short-term studies with clarithromycin reported reductions in IL-8 and neutrophil elastase, and modest decreases in neutrophils, although differences in macrolide, treatment duration, and endpoints limit direct comparison [42]. Macrolides also exhibit immunomodulatory activity—including calcineurin inhibition, downregulation of IL-5 transcription, and mTOR pathway blockade—which may influence both eosinophilic and neutrophilic inflammation [43]. Taken together, these findings indicate that macrolides may modulate multiple inflammatory pathways relevant to exacerbation risk, beyond bacterial eradication, and could also influence antiviral host defense. Although the antiviral mechanism of macrolides remains undefined, AMAZES showed parallel reductions in respiratory infections and asthma exacerbations, consistent with the established interplay between eosinophilic inflammation, impaired innate antiviral immunity, and virus-induced attacks [39].

##### Allergy and Pollution (Exposome)

In sensitized individuals, exposure to pollen, dust mites, or pet dander triggers IgE-mediated inflammation. This increases the risk of SAEs [44]. This immune reaction exacerbates airway inflammation and increases the risk of symptom flare-ups [45].

Exposure to air pollutants such as nitrogen dioxide (NO_2_), environmental tobacco smoke, and diesel fuel has been strongly associated with an increased risk of SAEs, as these substances promote oxidative stress and inflammation in the airways. Stress and dietary factors (low vitamin D levels and insufficient fish oil intake) have been shown to exacerbate asthma symptoms [30]. In line with these arguments, numerous studies have demonstrated that the season of the year represents an additional risk factor depending on the patient’s profile, due to varying risks of viral infections and the presence of aeroallergens throughout the year. Moreover, weather changes can trigger SAEs, as cold air and sudden temperature fluctuations may induce bronchoconstriction and worsen asthma control [46].

Climate change and extreme weather have been formally incorporated as risk modifiers in the 2025 GINA update [4]. The new section highlights that both extreme heat and extreme cold are associated with an increased need for urgent asthma care and a higher risk of exacerbations. These effects likely result from enhanced bronchial hyperresponsiveness and greater exposure to environmental triggers. GINA also notes that improved air quality—such as during COVID-19 lockdowns or during the strict pollution-control measures implemented for the Beijing 2008 Olympic Games—was accompanied by a marked reduction in asthma exacerbations, underscoring the impact of external environmental factors on disease instability [4].

Several studies indicate that the season of the year may act as a risk factor depending on the patient’s profile, due to changes in the prevalence of viral infections and aeroallergens throughout the year. Sensitisation and exposure to the fungal allergen *Alternaria alternata* (*A. alternata*) have been identified as risk factors for the onset of severe asthma symptoms, including life-threatening responses. In a study involving 11 patients who experienced episodes of respiratory arrest, 10 (91%) had positive skin prick test results for Alternaria alternata. In comparison, this proportion was 31% among 99 matched control subjects with asthma but without a history of respiratory arrest [47].

Beyond *Alternaria alternata*, other indoor and outdoor allergens can precipitate severe asthma worsening in sensitized individuals. High exposure to cat dander (Fel d 1) is strongly associated with bronchial hyperresponsiveness, loss of control, and increased risk of acute exacerbations in allergic asthma. Sensitization to dogs, dust mites (*Dermatophagoides pteronyssinus*, *D. farinae*), cockroach, and molds such as *Cladosporium* has also been linked to severe attacks, particularly when exposure is continuous or occurs in high concentrations [4]. In susceptible patients, even brief but intense exposure can trigger rapid-onset, severe exacerbations [48]. Conversely, several non-allergic triggers may worsen pulmonary function; β-blockers—especially non-selective agents—can induce bronchoconstriction and reduce bronchodilator response and should be avoided in patients with uncontrolled or severe asthma [49].

##### Nonsteroidal Anti-Inflammatory Drug-Exacerbated Respiratory Disease (NERD)

NERD, also known as aspirin-exacerbated respiratory disease (AERD), is a clinical syndrome defined by the triad of asthma, chronic rhinosinusitis with nasal polyps (CRSwNP), and hypersensitivity to nonsteroidal anti-inflammatory drugs (NSAIDs). It typically manifests in adulthood and is associated with eosinophilic airway inflammation and dysregulated arachidonic acid metabolism, including overproduction of cysteinyl leukotrienes and reduced prostaglandin E2 levels [50]. These patients often experience sudden and severe AEs after NSAID exposure, characterized by bronchospasm, increased mucus production, and respiratory distress that can be life-threatening. One study reported that NSAID intake was responsible of 5% of fatal or near-fatal asthma attack, 14% in sudden-onset and 3% in slow-onset groups [19].

These SAEs are typically non-IgE-mediated and are driven by enhanced leukotriene synthesis. Diagnosis is confirmed through aspirin challenge (nasal or oral) testing. Treatment includes NSAID avoidance, leukotriene receptor antagonists, aspirin desensitization, and biological therapy [51], which was shown to improve both upper and lower airway symptoms [50].

#### 3.2.2. Individual Factors and Comorbidities

Conditions such as obesity, gastroesophageal reflux disease (GERD), CRSwNP, and psychiatric disorders (e.g., anxiety and depression) have been linked to an elevated risk of exacerbations [52]. While there is no universal agreement on the specific interindividual factors that most strongly predispose individuals to exacerbations, numerous studies highlight that female sex, advanced age, a T2-inflamation profile, active smoking, metabolic dysfunction (including obesity and associated cardiovascular risk factors), and a low baseline forced expiratory volume in one second (FEV_1_) are statistically correlated with a higher exacerbation risk [53,54,55].

##### Obesity and Obesity Sleep Apnea (OSA)

Obesity and OSA are recognized comorbidities that significantly worsen asthma outcomes [56]. Obese individuals with asthma are at increased risk of frequent and severe exacerbations due to heightened airway inflammation and mechanical limitations [57,58]. OSA, in particular, is independently associated with poor asthma control and a higher frequency of severe exacerbations [59]. Additionally, recent evidence suggests that underweight individuals may also experience increased SAEs, potentially due to reduced respiratory muscle mass and nutritional deficiencies, although this relationship is less studied. Screening for OSA and assessing body weight status in asthmatic patients is essential for optimizing control and reducing acute episodes [60].

In obese asthma patients, inflammation is characterized by a distinct, often non-type 2 (T2-low) profile. This includes increased activation of innate immune responses with elevated neutrophilic airway inflammation and the involvement of macrophages and IL-6 signaling pathways. Adipose tissue-derived cytokines, particularly leptin, promote systemic inflammation and airway hyperresponsiveness, while adiponectin levels—typically anti-inflammatory—are reduced [61]. Additionally, obesity-related mechanical and metabolic factors contribute to a pro-inflammatory environment, further impairing lung function. Some patients may exhibit a mixed inflammatory pattern, with overlapping eosinophilic and neutrophilic features. This phenotype tends to respond poorly to corticosteroids and is associated with more severe symptoms, reduced quality of life, and increased exacerbation rates [56].

##### Gastroesophageal Reflux Disease (GERD)

GERD is a common comorbidity in asthma and is increasingly recognized as a trigger for SAEs. Studies show that up to 80% of patients with poorly controlled asthma may also experience GERD symptoms, even in the absence of typical heartburn or regurgitation. GERD can worsen asthma symptoms through micro-aspiration of gastric contents or vagally mediated reflex bronchoconstriction. Meta-analytic data confirms a significant association between GERD and increased SAE risk. Empirical treatment with acid-suppressive therapy, such as proton pump inhibitors, may improve asthma symptoms in a subset of patients with GERD-triggered asthma [62].

##### Psychiatric Disorders

Psychiatric comorbidities, particularly anxiety and depression, are common among individuals with asthma and are associated with worse disease control and more frequent exacerbations. Studies indicate that negative emotions and psychiatric symptoms can both precipitate and amplify asthma symptoms, contributing to functional respiratory complaints and reduced medication adherence. In adult populations, psychiatric illness is a significant predictor of acute SAEs, particularly in severe or treatment-resistant asthma cases [55].

#### 3.2.3. Respiratory Comorbidities and the Unified Airway

Respiratory comorbidities such as CRSwNP, bronchiectasis, and inducible laryngeal obstruction (ILO) frequently coexist with asthma and are associated with worsened symptom control and increased exacerbation risk [30].

CRSwNP, as part of the type 2 inflammatory spectrum, frequently coexists with asthma, contributing to eosinophilic inflammation, higher exacerbation rates, and worse disease control. These patients often experience poorer quality of life and may benefit from biologic therapies targeting the IL-4/IL-13 pathway [55].

Patients exhibiting features of both asthma and chronic obstructive pulmonary disease (ACO) typically present with fixed airflow obstruction, a history of smoking, and poor response to standard therapies. ACO is associated with higher exacerbation rates, lower lung function, and increased health care utilization. A personalized approach, often including triple inhaler therapy, namely, ICS, long-acting Beta2-agonist (LABA), and long-acting muscarinic antagonist (LAMA), is recommended [37,63].

ILO, previously known as vocal cord dysfunction, is increasingly recognized as a comorbidity or differential diagnosis in patients with refractory asthma symptoms. It involves paradoxical vocal cord closure during inspiration, leading to dyspnea, stridor, and poor response to bronchodilators. Diagnosis requires laryngoscopy during symptomatic episodes or exercise. ILO can coexist with dysfunctional breathing and significantly contributes to misdiagnosis and treatment resistance [64].

#### 3.2.4. Assessment of Treatment Adherence

Poor adherence to controller therapy or improper inhaler technique is a major contributor to SAEs and poor disease control. Both should be addressed in the management of EPA, as they are potential modifiable risk factors [65,66].

Multiple studies demonstrate that patients with high adherence (≥80%) to ICS have significantly fewer severe exacerbations. Similarly, real-world data show that lower prescription collection rates are associated with higher rescue therapy use, indicating more frequent acute episodes [67]. Objective measures of adherence, such as electronic monitoring and pharmacy refill records, tend to be more reliable than self-reporting, which often overestimates true use. Interestingly, frequent changes in inhaler devices are also linked to increased exacerbation rates, possibly due to confusion and improper technique. Interventions such as adherence monitoring, patient education, and simplification of regimens are key to improving asthma outcomes and reducing emergency visits.

#### 3.2.5. Behavioral, Socioeconomic, and Health-System Determinants

Behavioral and socioeconomic conditions strongly influence the likelihood of severe asthma exacerbations. In many countries, limited access to inhaled corticosteroid-containing medications remains a major obstacle, especially in low- and middle-income settings where asthma prevalence is rising and mortality is disproportionately high. Medication cost is one of the main drivers of nonadherence [68]. And when patients cannot reliably obtain ICS or ICS–LABA therapy, they are left to depend on short-acting bronchodilators, use incomplete controller regimens, or interrupt treatment altogether—patterns that substantially increase exacerbation risk. These patterns are exacerbated by low health literacy, incorrect inhaler technique, and delayed recognition of worsening symptoms, all of which contribute to preventable deterioration [69].

Structural inequities also limit access to specialist assessment and advanced therapies. Long waiting times, fragmented care pathways, and restricted implementation of biomarker-guided evaluation delay the identification of high-risk phenotypes. Social vulnerability—such as unstable employment, housing insecurity, and chronic stress—adds further exposure to indoor pollutants, environmental tobacco smoke, and occupational irritants, amplifying airway inflammation and exacerbation frequency [70]. Global efforts to improve the availability of affordable, high-quality ICS–formoterol inhalers and to reduce dependence on propellant-based SABA devices are essential to address these disparities. Ensuring continuous supply chains, expanding access to guideline-based treatment, and adapting care strategies to resource-limited contexts are critical steps to reduce preventable severe exacerbations worldwide [4].

## 4. Discussion

### 4.1. Exacerbation Approach

AEs are most commonly classified by severity, as this guides immediate management [71]. Both the GINA [4] and the Spanish Guideline for the Management of Asthma (GEMA) [72] categorize attacks as mild, moderate, severe, or life-threatening, based on clinical presentation in the emergency department. An AE’s severity determines the appropriate care pathway [71].

#### 4.1.1. Current Standard of Care

Clinical guidelines identify three main treatments for acute AEs: rapid-acting bronchodilators, oral corticosteroids (OCSs), and oxygen therapy as needed. Both guidelines recommend short-acting β2-agonist (SABA) like salbutamol as first-line therapy; for severe cases, adding a LAMA such as ipratropium bromide is advised. Systemic corticosteroids, typically oral prednisone 40–50 mg daily for 5–7 days, reduce airway inflammation and prevent relapse, with intravenous options for selected severe cases. Oxygen therapy, though not pharmacological, is essential when hypoxemia occurs [4,72].

While the benefits of bronchodilator therapy are well-established through its mechanism of action, corticosteroid treatment—also routinely indicated in all SAE—has limited evidence supporting their actual efficacy across the various exacerbation phenotypes.

#### 4.1.2. Emerging Management Perspectives

Over the past few years, advances in the understanding of asthma’s complex pathophysiology have led to a paradigm transition in the management of acute exacerbations. Rather than applying a uniform treatment approach, there is growing emphasis on tailoring interventions based on distinct clinical phenotypes and underlying biological (endotype) mechanisms. This shift reflects the recognition that asthma exacerbations are not a homogeneous entity and that more personalized strategies may enhance therapeutic efficacy and minimize unnecessary exposure to systemic treatments [73].

One promising direction in this field is the incorporation of T2 inflammatory biomarker-guided approaches that enable the stratification of patients. Blood eosinophil count (BEC) and fractional exhaled nitric oxide (FeNO) offer complementary insights into distinct compartments of T2 inflammation: BEC reflects systemic eosinophilic activity driven by interleukin-5, while FeNO is a surrogate of airway epithelial activation through IL-4/IL-13 pathways [73]. Unlike symptom scores or spirometric indices, which correlate poorly with exacerbation risk, these biomarkers have demonstrated consistent prognostic value across asthma severities [74]. Recent studies have shown that elevated levels of BEC and/or FeNO are associated with a substantially increased risk of severe exacerbations, and more importantly, this risk appears modifiable with targeted anti-inflammatory treatment—including ICS in mild asthma and biologics in more severe phenotypes. Conversely, patients with low biomarker levels often exhibit minimal response to such interventions, raising concerns about overtreatment and iatrogenic harm [75]. One illustrative example is the prototype ORACLE scale, which stratifies exacerbation risk and estimates treatment benefit based on BEC and FeNO thresholds, showing promising alignment between predicted and observed outcomes in clinical trials [74]. These findings underscore the theragnostic potential of T2 biomarkers to not only predict future risk but also guide individualized therapy. Incorporating biomarker-based stratification into routine care could enhance the precision of acute asthma management by identifying patients most likely to benefit from anti-inflammatory therapies while minimizing unnecessary systemic exposure in low-risk individuals [76,77].

Recent efforts to routinely perform chest computed tomography (CT) in patients with severe, uncontrolled asthma to detect pathological changes have highlighted a patient phenotype characterized by asthma–bronchiectasis overlap. Bronchiectasis, characterized by permanent bronchial dilation and chronic infection, occurs in up to 30% of patients with longstanding or severe asthma. It is associated with increased sputum production, airway colonization by pathogens, and heightened inflammatory response, contributing to more frequent and severe AEs. Its coexistence often indicates a specific phenotype with worse lung function and greater need for targeted antimicrobial and airway clearance therapies [78].

**Figure 2:**
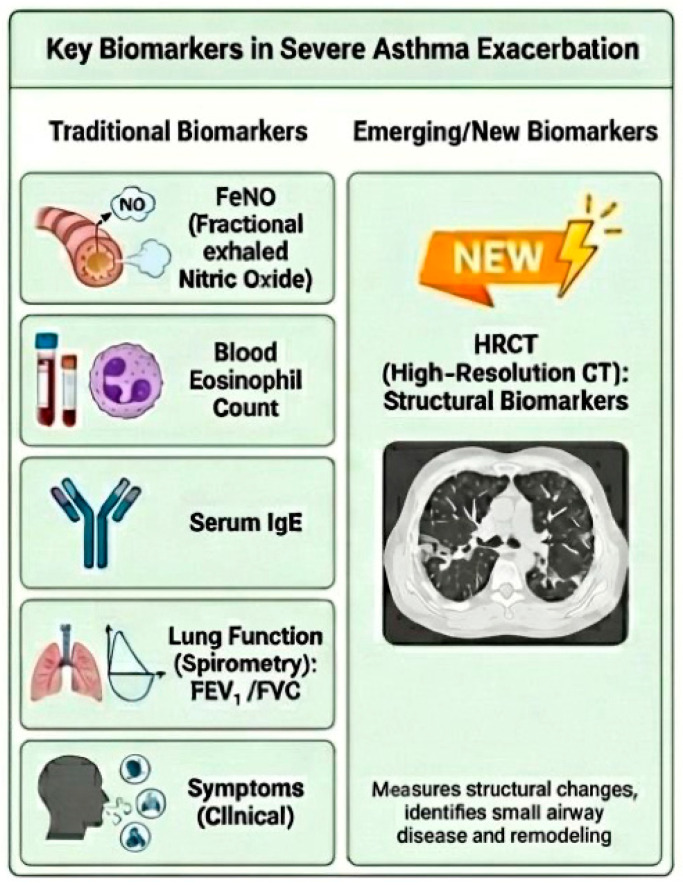
Key biomarkers in severe asthma exacerbation. NO: nitric oxide; FeNO: fractional exhaled nitric oxide; IgE: immunoglobulin E; FEV1: forced expiratory volume at first second; FVC: forced vital capacity; HRCT: high-resolution computed tomography.

Imaging studies show that mucus plugs are more common in asthma patients than in the general population. Higher mucus scores are linked to poorer baseline lung function and an increased risk of exacerbations [79]. Figure 2 summarizes the key traditional and emerging biomarkers previously discussed as central components of severe asthma exacerbation phenotyping.

A recently proposed approach focuses on characterizing individuals with airway diseases by identifying treatable traits—modifiable elements that impact symptoms and clinical outcomes [80]. While this approach has shown considerable promise in chronic obstructive pulmonary disease (COPD) and older patients with airway diseases, its applicability to severe asthma remains uncertain.

Recent studies have shown that individual treatable traits are found more often in severe asthma compared to non-severe forms [64]. Some of the most common traits found include eosinophilic inflammation, chronic airway infection (including bronchiectasis and pathogenic colonization), obesity, OSA, GERD, dysfunctional breathing, psychiatric disorders, and ILO. Each of these traits may contribute independently to exacerbation frequency and poor asthma control. Addressing them can improve outcomes beyond standard pharmacologic treatment [81]. Latest evidence shows that the treatable-traits approach is clinically relevant, particularly when examining factors that limit the probability of achieving remission. A recent large meta-analysis demonstrated that several comorbidities—obesity (OR 0.41), depression (OR 0.38), OSA (OR 0.48), ACO (OR 0.45), and osteoporosis (OR 0.48)—substantially decrease the likelihood of clinical remission in patients receiving biologics [82]. These results indicate that identifying and treating asthma together with coexisting treatable traits may be a beneficial therapeutic approach, particularly in patients with multiple comorbidities who experience recurrent or severe exacerbations, highlighting the need for early recognition and targeted management. The PRISMA study [83,84] examined the effectiveness of OCS in managing asthma exacerbations (AEs) given the varied inflammatory profiles involved. The researchers assessed whether T2 inflammatory biomarkers—BEC and FeNO—could predict response to corticosteroid therapy during severe asthma attacks. AE was categorized into T2-Low/Low, T2-Mid, and T2-High/High groups based on biomarker levels. Only the T2-High/High group showed significant improvement in post-bronchodilator FEV_1_, while gains were minimal for T2-Low/Low cases. Adverse events from OCS occurred across all groups, indicating potential overtreatment in those with low biomarkers. The study supports using dual biomarkers to guide OCS use in acute asthma, promoting more targeted and safer treatment.

Although the emergence of biologic therapies targeting type 2 inflammation has reshaped the therapeutic landscape of severe asthma, including its most critical presentations, evidence supporting their use during acute severe asthma exacerbations remains limited. The main randomized controlled evidence comes from the phase 2 Acute exacerbations treated with BenRAlizumab (ABRA) trial. In ABRA, adults presenting to urgent care clinics or emergency departments with eosinophilic acute exacerbations of asthma or COPD and blood eosinophil counts ≥300 cells/µL were randomly assigned to receive a 5-day course of oral prednisolone, a single 100 mg subcutaneous dose of benralizumab, or the combination. Benralizumab-containing regimens significantly reduced the proportion of treatment failures over 90 days compared with prednisolone alone (from 74% to 45%; odds ratio 0.26, 95% CI 0.13–0.56) and led to more rapid improvement in symptom scores at 28 days. However, ABRA was a relatively small, phase 2 study, used a higher benralizumab dose than that approved for maintenance treatment in severe eosinophilic asthma, and enrolled a highly selected eosinophilic population [85].

**Figure 3:**
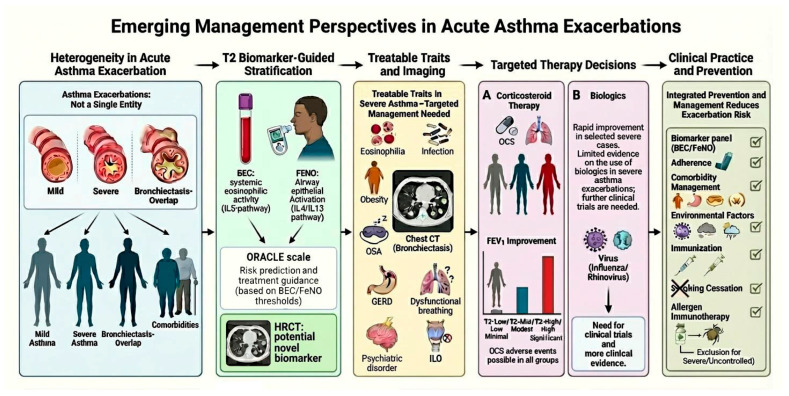
Emerging management perspectives in acute asthma exacerbations. T2: type 2 inflammation; BEC: blood eosinophil count; FeNO: fractional exhaled nitric oxide; IL: interleukin; HRCT: high-resolution computed tomography; OSA: obstructive sleep apnea; CT: computed tomography; GERD: gastroesophageal reflux disease; ILO: inducible laryngeal obstruction; OCS: oral corticosteroid; FEV1: forced expiratory volume at first second.

Consistent with this mechanism, mepolizumab has also shown rapid and clinically meaningful improvement in isolated case series of life-threatening eosinophilic exacerbations, supporting the plausibility of IL-5-directed therapy in the acute setting [86]. Reports of successful use of tezepelumab—a thymic stromal lymphopoietin (TSLP) inhibitor—in near-fatal viral-triggered exacerbations further reinforce the hypothesis that upstream blockade of alarmin-mediated inflammation could attenuate epithelial-driven cytokine responses in the acute phase [87]. These therapeutic effects support the hypothesis that biologics may attenuate virus-induced inflammation and airway remodeling during exacerbations, without compromising antiviral immunity [88]. Nevertheless, these findings have not yet translated into regulatory approval or into changes in current guideline-recommended acute care, where systemic corticosteroids and bronchodilators remain the cornerstone of management, and the initiation of biologics during an acute severe exacerbation is still regarded as experimental. Emerging management perspectives in acute asthma exacerbations are summarized in Figure 3.

### 4.2. Practical Implications for Clinicians

Although severe asthma exacerbations arise from heterogeneous mechanisms, several practical considerations can enhance clinical decision-making. First, systematic assessment of T2 biomarkers (BEC and FeNO) can identify patients more likely to benefit from anti-inflammatory therapies during acute episodes and help avoid unnecessary systemic corticosteroids in low-biomarker phenotypes. Second, routine verification of inhaler technique, adherence, and modifiable comorbidities—particularly obesity, OSA, GERD, and smoking—remains essential, as these factors substantially influence exacerbation risk and therapeutic response. Third, recent GINA updates emphasize the relevance of environmental modifiers, including viral circulation peaks, air quality fluctuations, and extreme weather events, which should inform anticipatory advice in high-risk patients [4]. Finally, clinicians should recognize exacerbation-prone asthma as a distinct phenotype requiring intensified follow-up and, when appropriate, early consideration of biologic therapy. Incorporating these elements into everyday practice may reduce overtreatment, improve precision during acute care, and ultimately lower future exacerbation burden.

### 4.3. Prevention of Exacerbations

Preventing severe exacerbations requires addressing modifiable factors with proven clinical benefit. Annual influenza vaccination reduces the risk of virus-induced attacks and is recommended for all adults with asthma [4]. Pneumococcal vaccination is also advised, particularly in older patients and those with comorbidities, to lower the risk of invasive disease that can worsen asthma control [81].

Smoking cessation is a key intervention. Active smoking impairs corticosteroid responsiveness, increases airway inflammation, and markedly raises exacerbation risk [82]. Brief interventions, behavioral support, and pharmacological therapy improve quit rates and should be systematically offered [83].

In allergic asthma, allergen immunotherapy can reduce symptoms and exacerbations in selected patients with clear sensitization and exposure. Its benefit is most consistent for pollen-driven and dust-mite-driven disease. Careful phenotyping and adherence assessment are essential to identify candidates who are likely to respond. Allergen immunotherapy is not recommended in patients with uncontrolled severe asthma, as it can precipitate severe exacerbations or even life-threatening reactions [4]. Adequate symptom control and risk assessment are mandatory before initiating treatment.

These preventive measures complement pharmacological management and address upstream drivers of acute episodes. Their systematic implementation remains uneven and represents an important area for improvement in clinical practice.

This review has several limitations. First, it is a narrative, non-systematic review; therefore, the search strategy was not exhaustive, and no predefined inclusion or exclusion criteria were applied, introducing a risk of selection and confirmation bias. Second, the studies included in this review are highly heterogeneous in their definitions of exacerbation severity, asthma severity, and clinical phenotypes, limiting the comparability of findings across populations. Also, no formal assessment of risk of bias was performed for the included studies, and many of the associations described may be influenced by unmeasured confounding or diagnostic variability, particularly in infection-related and comorbidity-related exacerbations. Moreover, the evidence supporting the use of biologics during acute severe exacerbations is based almost exclusively on case reports and small case series. These observations cannot establish treatment efficacy or causality and have limited generalizability. Finally, most available data come from tertiary or specialist cohorts in high-income settings, which restricts the external validity of the conclusions and their applicability to primary care or low-resource environments.

## 5. Conclusions

Despite substantial advances in asthma care, severe exacerbations remain common and clinically impactful. Their persistence reflects the complex interplay between environmental triggers, individual patient traits, and underlying inflammatory endotypes, which is not fully captured by current severity-based strategies. Moving forward, integrating type 2 biomarkers such as BEC and FeNO into acute-care decision-making, and rigorously testing biomarker- and phenotype-guided approaches, will be essential to reduce the burden of severe exacerbations and to achieve more truly individualized care. The potential role of biologics in acute severe exacerbations remains experimental and requires further high-quality evaluation before it can be incorporated into routine practice.

In this context, further studies are needed to validate specific biomarker-guided and phenotype-based approaches in diverse clinical settings, explore their applicability during acute episodes, and assess their impact on long-term outcomes.

## Data Availability

No new data were created or analyzed in this study. Data sharing is not applicable to this article.

## Abbreviations

The following abbreviations are used in this manuscript:

SAE	Severe asthma exacerbation
T2	Type 2
BEC	Blood eosinophil count
FeNO	Fractional exhaled nitric oxide
GINA	Global Initiative for Asthma
EPA	Exacerbation-prone asthma
ICS	Inhaled corticosteroid
OSA	Obesity sleep apnea
NERD	Nonsteroidal anti-inflammatory drug-exacerbated respiratory disease
T2	Type-2
IL	Interleukin
NET	Neutrophil extracellular trap
FEV_1_	Forced expiratory volume in one second
IgE	Immunoglobulin E
GERD	Gastroesophageal reflux disease
CRSwNP	Chronic rhinosinusitis with nasal polyps
ILO	Inducible laryngeal obstruction
ACO	Asthma and chronic obstructive disease
LABA	Long-acting Beta2-agonist
LAMA	Long-acting muscarinic antagonist
GEMA	Spanish Guideline for the Management of Asthma
OCS	Oral corticosteroid
SABA	Short-acting Beta2-agonist
CT	Computed tomography
COPD	Chronic obstructive pulmonary disease
TSLP	Thymic stromal lymphopoietin

## References

[1] Fuhlbrigge A., Peden D., Apter A.J., Boushey H.A., Camargo C.A., Gern J., Heymann P.W., Martinez F.D., Mauger D., Teague W.G. (2012). Asthma Outcomes: Exacerbations. J. Allergy Clin. Immunol..

[2] Ilmarinen P., Juboori H., Tuomisto L.E., Niemelä O., Sintonen H., Kankaanranta H. (2019). Effect of Asthma Control on General Health-Related Quality of Life in Patients Diagnosed with Adult-Onset Asthma. Sci. Rep..

[3] Luskin A.T., Chipps B.E., Rasouliyan L., Miller D.P., Haselkorn T., Dorenbaum A. (2014). Impact of Asthma Exacerbations and Asthma Triggers on Asthma-Related Quality of Life in Patients with Severe or Difficult-to-Treat Asthma. J. Allergy Clin. Immunol. Pract..

[4] Global Initiative for Asthma (2025). Global Strategy for Asthma Management and Prevention. http://www.ginasthma.org.

[5] Lee T.Y., Price D., Yadav C.P., Roy R., Lim L.H.M., Wang E., Wechsler M.E., Jackson D.J., Busby J., Heaney L.G. (2024). International Variation in Severe Exacerbation Rates in Patients with Severe Asthma. CHEST.

[6] Jackson D.J., McDonald V.M., Pavord I.D. (2025). Asthma.

[7] Suruki R.Y., Daugherty J.B., Boudiaf N., Albers F.C. (2017). The Frequency of Asthma Exacerbations and Healthcare Utilization in Patients with Asthma from the UK and USA. BMC Pulm. Med..

[8] Denlinger L.C., Heymann P., Lutter R., Gern J.E. (2020). Exacerbation-Prone Asthma. J. Allergy Clin. Immunol. Pract..

[9] FitzGerald J.M., Barnes P.J., Chipps B.E., Jenkins C.R., O’Byrne P.M., Pavord I.D., Reddel H.K. (2020). The Burden of Exacerbations in Mild Asthma: A Systematic Review. ERJ Open Res..

[10] Kim M.-A., Shin S.-W., Park J.-S., Uh S.-T., Chang H.S., Bae D.-J., Cho Y.S., Park H.-S., Yoon H.J., Choi B.W. (2017). Clinical Characteristics of Exacerbation-Prone Adult Asthmatics Identified by Cluster Analysis. Allergy Asthma Immunol. Res..

[11] Wang E., Wechsler M.E., Tran T.N., Heaney L.G., Jones R.C., Menzies-Gow A.N., Busby J., Jackson D.J., Pfeffer P.E., Rhee C.K. (2020). Characterization of Severe Asthma Worldwide. Chest.

[12] Czira A., Turner M., Martin A., Hinds D., Birch H., Gardiner F., Zhang S. (2022). A Systematic Literature Review of Burden of Illness in Adults with Uncontrolled Moderate/Severe Asthma. Respir. Med..

[13] Fleming L. (2018). Asthma Exacerbation Prediction: Recent Insights. Curr. Opin. Allergy Clin. Immunol..

[14] Yii A.C.A., Tan J.H.Y., Lapperre T.S., Chan A.K.W., Low S.Y., Ong T.H., Tan K.L., Chotirmall S.H., Sterk P.J., Koh M.S. (2017). Long-term Future Risk of Severe Exacerbations: Distinct 5-year Trajectories of Problematic Asthma. Allergy.

[15] Loymans R.J.B., Debray T.P.A., Honkoop P.J., Termeer E.H., Snoeck-Stroband J.B., Schermer T.R.J., Assendelft W.J.J., Timp M., Chung K.F., Sousa A.R. (2018). Exacerbations in Adults with Asthma: A Systematic Review and External Validation of Prediction Models. J. Allergy Clin. Immunol. Pract..

[16] Singh D., Oosterholt S., Pavord I., Garcia G., Abhijith P.G., Della Pasqua O. (2023). Understanding the Clinical Implications of Individual Patient Characteristics and Treatment Choice on the Risk of Exacerbation in Asthma Patients with Moderate–Severe Symptoms. Adv. Ther..

[17] Lane S.J., Petersen H., Seltzer J.M., Blanchette C.M., Navaratnam P., Allen-Ramey F., Fuhlbrigge A. (2013). Moderate Symptom-Based Exacerbations as Predictors of Severe Claims-Based Exacerbations in Asthma. J. Asthma.

[18] Barr R.G., Woodruff P.G., Clark S., Camargo C.A. (2000). On Behalf of the Multicenter Airway Research Collaboration (Marc) Investigators Sudden-Onset Asthma Exacerbations: Clinical Features, Response to Therapy, and 2-Week Follow-Up. Eur. Respir. J..

[19] Plaza V., Serrano J., Picado C., Sanchis J. (2002). Frequency and Clinical Characteristics of Rapid-Onset Fatal and near-Fatal Asthma. Eur. Respir. J..

[20] Serrano J., Plaza V., Sureda B., De Pablo J., Picado C., Bardagí S., Lamela J., Sanchis J. (2006). Alexithymia: A Relevant Psychological Variable in near-Fatal Asthma. Eur. Respir. J..

[21] Picado C., Castillo J., Montserrat J., Agusti-Vidal A. (1989). Aspirin-Intolerance as a Precipitating Factor of Life-Threatening Attacks of Asthma Requiring Mechanical Ventilation. Eur. Respir. J..

[22] Martinez-Moragón E., Plaza V., Serrano J., Picado C., Galdiz J.B., López-Viña A., Sanchis J. (2004). Near-Fatal Asthma Related to Menstruation. J. Allergy Clin. Immunol..

[23] Picado C. (1996). Classification of Severe Asthma Exacerbations: A Proposal. Eur. Respir. J..

[24] Paredes López M., Osorio Trujillo J., Rodríguez Lobato E., Cedillo Huerta H.E., de la Fuente García A., Lafuente J.M., Riudor Guri A., Albacar Ingla N., Picado Vallés C., Arismendi Núñez E. Agudización asmática grave en la UCI: Nuestra experiencia en el Hospital Clínic de Barcelona. Proceedings of the 58.º Congreso de la Sociedad Española de Neumología y Cirugía Torácica (SEPAR).

[25] Thien F., Davies J.M., Douglass J.A., Hew M. (2025). Thunderstorm Asthma: Current Perspectives and Emerging Trends. J. Allergy Clin. Immunol. Pract..

[26] Picado C. (1992). Barcelona’s Asthma Epidemics: Clinical Aspects and Intriguing Findings. Thorax.

[27] Johnston N.W. (2006). Asthma Exacerbations·1: Epidemiology. Thorax.

[28] Busse W.W., Kraft M., Rabe K.F., Deniz Y., Rowe P.J., Ruddy M., Castro M. (2021). Understanding the Key Issues in the Treatment of Uncontrolled Persistent Asthma with Type 2 Inflammation. Eur. Respir. J..

[29] Castillo J.R., Peters S.P., Busse W.W. (2017). Asthma Exacerbations: Pathogenesis, Prevention, and Treatment. J. Allergy Clin. Immunol. Pract..

[30] Muñoz X., Álvarez-Puebla M.J., Arismendi E., Arochena L., Del Pilar Ausín M., Barranco P., Bobolea I., Cañas J.A., Cardaba B., Crespo A. (2018). The MEGA Project: A Study of the Mechanisms Involved in the Genesis and Disease Course of Asthma. Asthma Cohort Creation and Long-Term Follow-Up. Arch. Bronconeumol..

[31] Ojanguren I., Satia I., Usmani O.S. (2022). The Role of Viral Infections on Severe Asthma Exacerbations: Present and Future. Arch. Bronconeumol..

[32] Hewitt R., Farne H., Ritchie A., Luke E., Johnston S.L., Mallia P. (2016). The Role of Viral Infections in Exacerbations of Chronic Obstructive Pulmonary Disease and Asthma. Ther. Adv. Respir. Dis..

[33] Cortjens B., De Boer O.J., De Jong R., Antonis A.F., Sabogal Piñeros Y.S., Lutter R., Van Woensel J.B., Bem R.A. (2016). Neutrophil Extracellular Traps Cause Airway Obstruction during Respiratory Syncytial Virus Disease. J. Pathol..

[34] Dunican E.M., Elicker B.M., Gierada D.S., Nagle S.K., Schiebler M.L., Newell J.D., Raymond W.W., Lachowicz-Scroggins M.E., Di Maio S., Hoffman E.A. (2018). Mucus Plugs in Patients with Asthma Linked to Eosinophilia and Airflow Obstruction. J. Clin. Investig..

[35] Ji T., Li H. (2023). T-Helper Cells and Their Cytokines in Pathogenesis and Treatment of Asthma. Front. Immunol..

[36] Jackson D.J., Johnston S.L. (2010). The Role of Viruses in Acute Exacerbations of Asthma. J. Allergy Clin. Immunol..

[37] Kurai D., Saraya T., Ishii H., Takizawa H. (2013). Virus-Induced Exacerbations in Asthma and COPD. Front. Microbiol..

[38] Bourdin A., Bjermer L., Brightling C., Brusselle G.G., Chanez P., Chung K.F., Custovic A., Diamant Z., Diver S., Djukanovic R. (2019). ERS/EAACI Statement on Severe Exacerbations in Asthma in Adults: Facts, Priorities and Key Research Questions. Eur. Respir. J..

[39] Lieberman D., Lieberman D., Printz S., Ben-Yaakov M., Lazarovich Z., Ohana B., Friedman M.G., Dvoskin B., Leinonen M., Boldur I. (2003). Atypical Pathogen Infection in Adults with Acute Exacerbation of Bronchial Asthma. Am. J. Respir. Crit. Care Med..

[40] Gibson P.G., Yang I.A., Upham J.W., Reynolds P.N., Hodge S., James A.L., Jenkins C., Peters M.J., Marks G.B., Baraket M. (2017). Effect of Azithromycin on Asthma Exacerbations and Quality of Life in Adults with Persistent Uncontrolled Asthma (AMAZES): A Randomised, Double-Blind, Placebo-Controlled Trial. Lancet.

[41] Iikura M., Hojo M., Koketsu R., Watanabe S., Sato A., Chino H., Ro S., Masaki H., Hirashima J., Ishii S. (2015). The Importance of Bacterial and Viral Infections Associated with Adult Asthma Exacerbations in Clinical Practice. PLoS ONE.

[42] Simpson J.L., Powell H., Boyle M.J., Scott R.J., Gibson P.G. (2008). Clarithromycin Targets Neutrophilic Airway Inflammation in Refractory Asthma. Am. J. Respir. Crit. Care Med..

[43] Ratzinger F., Haslacher H., Poeppl W., Hoermann G., Kovarik J.J., Jutz S., Steinberger P., Burgmann H., Pickl W.F., Schmetterer K.G. (2014). Azithromycin Suppresses CD4+ T-Cell Activation by Direct Modulation of mTOR Activity. Sci. Rep..

[44] Del Giacco S.R., Bakirtas A., Bel E., Custovic A., Diamant Z., Hamelmann E., Heffler E., Kalayci Ö., Saglani S., Sergejeva S. (2017). Allergy in Severe Asthma. Allergy.

[45] Messaoud-Nacer Y., Culerier E., Rose S., Maillet I., Boussad R., Veront C., Savigny F., Malissen B., Radzikowska U., Sokolowska M. (2025). STING-dependent Induction of Neutrophilic Asthma Exacerbation in Response to House Dust Mite. Allergy.

[46] Oosterholt S., Pavord I.D., Brusselle G., Yorgancıoğlu A., Pitrez P.M., Pg A., Teli C., Della Pasqua O. (2023). Modelling ASthma TrEatment Responses (MASTER): Effect of Individual Patient Characteristics on the Risk of Exacerbation in Moderate or Severe Asthma: A Time-to-event Analysis of Randomized Clinical Trials. Brit. J. Clin. Pharma..

[47] O’Hollaren M.T., Yunginger J.W., Offord K.P., Somers M.J., O’Connell E.J., Ballard D.J., Sachs M.I. (1991). Exposure to an Aeroallergen as a Possible Precipitating Factor in Respiratory Arrest in Young Patients with Asthma. N. Engl. J. Med..

[48] Custovic A., Simpson A. (2012). The Role of Inhalant Allergens in Allergic Airways Disease. J. Investig. Allergol. Clin. Immunol..

[49] Morales D.R., Lipworth B.J., Donnan P.T., Jackson C., Guthrie B. (2017). Respiratory Effect of Beta-Blockers in People with Asthma and Cardiovascular Disease: Population-Based Nested Case Control Study. BMC Med..

[50] Narayanankutty A., Reséndiz-Hernández J.M., Falfán-Valencia R., Teran L.M. (2013). Biochemical Pathogenesis of Aspirin Exacerbated Respiratory Disease (AERD). Clin. Biochem..

[51] Wangberg H., Spierling Bagsic S.R., Osuna L., White A.A. (2022). Appraisal of the Real-World Effectiveness of Biologic Therapies in Aspirin-Exacerbated Respiratory Disease. J. Allergy Clin. Immunol. Pract..

[52] Boulay M.-È., Pruneau-Pomerleau C., Villeneuve H., Deschesnes F., Ringuette L., Boulet L.-P. (2018). Comparative Features of Asthma with Frequent or Infrequent Exacerbations: A Longitudinal Study of Retrospective and Prospective Events. J. Asthma.

[53] O’Byrne P.M., Pedersen S., Lamm C.J., Tan W.C., Busse W.W. (2009). Severe Exacerbations and Decline in Lung Function in Asthma. Am. J. Respir. Crit. Care Med..

[54] Tay T.R., Radhakrishna N., Hore-Lacy F., Smith C., Hoy R., Dabscheck E., Hew M. (2016). Comorbidities in Difficult Asthma Are Independent Risk Factors for Frequent Exacerbations, Poor Control and Diminished Quality of Life. Respirology.

[55] Domínguez-Ortega J., Luna-Porta J., Olaguibel J., Barranco P., Arismendi E., Barroso B., Betancor D., Bobolea I., Caballero M., Cárdaba B. (2023). Exacerbations Among Patients With Asthma Are Largely Dependent on the Presence of Multimorbidity. J. Investig. Allergol. Clin..

[56] Arismendi E., Bantulà M., Picado C. (2023). Obese Asthma Syndrome: Much Work to Do. Arch. Bronconeumol..

[57] Dixon A.E., Clerisme-Beaty E.M., Sugar E.A., Cohen R.I., Lang J.E., Brown E.D., Richter J.E., Irvin C.G., Mastronarde J.G. (2011). Effects of Obstructive Sleep Apnea and Gastroesophageal Reflux Disease on Asthma Control in Obesity. J. Asthma.

[58] Becerra M.B., Becerra B.J., Teodorescu M. (2016). Healthcare Burden of Obstructive Sleep Apnea and Obesity among Asthma Hospitalizations: Results from the U.S.-Based Nationwide Inpatient Sample. Respir. Med..

[59] Wang Y., Liu K., Hu K., Yang J., Li Z., Nie M., Dong Y., Huang H., Chen J. (2016). Impact of Obstructive Sleep Apnea on Severe Asthma Exacerbations. Sleep Med..

[60] Staggers K.A., Minard C., Byers M., Helmer D.A., Wu T.D. (2023). Metabolic Dysfunction, Triglyceride-Glucose Index, and Risk of Severe Asthma Exacerbation. J. Allergy Clin. Immunol. Pract..

[61] Bantulà M., Tubita V., Roca-Ferrer J., Mullol J., Valero A., Bobolea I., Pascal M., De Hollanda A., Vidal J., Picado C. (2022). Differences in Inflammatory Cytokine Profile in Obesity-Associated Asthma: Effects of Weight Loss. J. Clin. Med..

[62] Mallah N., Turner J.M., González-Barcala F., Takkouche B. (2022). Gastroesophageal Reflux Disease and Asthma Exacerbation: A Systematic Review and Meta-analysis. Pediatr. Allergy Immunol..

[63] Laitano R., Calzetta L., Motta E., Puxeddu E., Rogliani P. (2024). Role of Exosomes in Exacerbations of Asthma and COPD: A Systematic Review. Front. Mol. Biosci..

[64] McDonald V.M., Hiles S.A., Godbout K., Harvey E.S., Marks G.B., Hew M., Peters M., Bardin P.G., Reynolds P.N., Upham J.W. (2019). Treatable Traits Can Be Identified in a Severe Asthma Registry and Predict Future Exacerbations. Respirology.

[65] Engelkes M., Janssens H.M., De Jongste J.C., Sturkenboom M.C.J.M., Verhamme K.M.C. (2015). Medication Adherence and the Risk of Severe Asthma Exacerbations: A Systematic Review. Eur. Respir. J..

[66] Gutiérrez F.J.Á., Galván M.F., Gallardo J.F.M., Mancera M.B., Romero B.R., Falcón A.R. (2017). Predictive Factors for Moderate or Severe Exacerbations in Asthma Patients Receiving Outpatient Care. BMC Pulm. Med..

[67] Koh Y.L.E., Chua K.Y.K., Ng D.X., Aau W.K., Tan N.C. (2025). Assessing Medication Adherence in Adults with Asthma and Its Effect on Rescue Therapy for Exacerbations. Front. Pharmacol..

[68] Stolbrink M., Chinouya M.J., Jayasooriya S., Nightingale R., Evans-Hill L., Allan K., Allen H., Balen J., Beacon T., Bissell K. (2022). Improving Access to Affordable Quality-Assured Inhaled Medicines in Low- and Middle-Income Countries. Int. J. Tuberc. Lung Dis..

[69] Meghji J., Mortimer K., Agusti A., Allwood B.W., Asher I., Bateman E.D., Bissell K., Bolton C.E., Bush A., Celli B. (2021). Improving Lung Health in Low-Income and Middle-Income Countries: From Challenges to Solutions. Lancet.

[70] Clougherty J.E., Kubzansky L.D. (2009). A Framework for Examining Social Stress and Susceptibility to Air Pollution in Respiratory Health. Environ. Health Perspect..

[71] Ramsahai J.M., Hansbro P.M., Wark P.A.B. (2019). Mechanisms and Management of Asthma Exacerbations. Am. J. Respir. Crit. Care Med..

[72] (2024). Guía Española Para El Manejo Del Asma. https://www.semg.es/images/2024/documentos/GEMA_54.pdf.

[73] Couillard S., Pavord I., Hoyte F., Siddiqui S., Martin N., Menzies-Gow A., Lommatzsch M. (2025). “Treat-to-Target”: A Call for Earlier Targeted Intervention in Asthma. J. Allergy Clin. Immunol. Pract..

[74] Meulmeester F.L., Mailhot-Larouche S., Celis-Preciado C., Lemaire-Paquette S., Ramakrishnan S., Wechsler M.E., Brusselle G., Corren J., Hardy J., Diver S.E. (2025). Inflammatory and Clinical Risk Factors for Asthma Attacks (ORACLE2): A Patient-Level Meta-Analysis of Control Groups of 22 Randomised Trials. Lancet Respir. Med..

[75] Howell I., Noble J., Howell A., Morgan C., Logan J., Miller S., Chaudhuri R., Russell R.E.K., Bafadhel M., Beasley R. (2025). The Risk–Benefit Balance of Oral Corticosteroid Treatment for Asthma Attacks: A Discrete Choice Experiment of Patients and Healthcare Professionals in the UK and New Zealand. Respirology.

[76] Couillard S., Do W.I.H., Beasley R., Hinks T.S.C., Pavord I.D. (2022). Predicting the Benefits of Type-2 Targeted Anti-Inflammatory Treatment with the Prototype Oxford Asthma Attack Risk Scale (ORACLE). ERJ Open Res..

[77] Couillard S., Laugerud A., Jabeen M., Ramakrishnan S., Melhorn J., Hinks T., Pavord I. (2022). Derivation of a Prototype Asthma Attack Risk Scale Centred on Blood Eosinophils and Exhaled Nitric Oxide. Thorax.

[78] García-Clemente M., Enríquez-Rodríguez A.I., Iscar-Urrutia M., Escobar-Mallada B., Arias-Guillén M., López-González F.J., Madrid-Carbajal C., Pérez-Martínez L., Gonzalez-Budiño T. (2020). Severe Asthma and Bronchiectasis. J. Asthma.

[79] Yoshida Y., Takaku Y., Nakamoto Y., Takayanagi N., Yanagisawa T., Takizawa H., Kurashima K. (2020). Changes in Airway Diameter and Mucus Plugs in Patients with Asthma Exacerbation. PLoS ONE.

[80] Ten Brinke A., Sterk P.J., Masclee A.A.M., Spinhoven P., Schmidt J.T., Zwinderman A.H., Rabe K.F., Bel E.H. (2005). Risk Factors of Frequent Exacerbations in Difficult-to-Treat Asthma. Eur. Respir. J..

[81] Tay T.R., Hew M. (2018). Comorbid “Treatable Traits” in Difficult Asthma: Current Evidence and Clinical Evaluation. Allergy.

[82] Shackleford A., Heaney L.G., Redmond C., McDowell P.J., Busby J. (2025). Clinical Remission Attainment, Definitions, and Correlates among Patients with Severe Asthma Treated with Biologics: A Systematic Review and Meta-Analysis. Lancet Respir. Med..

[83] Celis-Preciado C., Leclerc S., Duval M., Cliche D.O., Brazeau L., Vézina F.-A., Dussault M., Larivée P., Lemaire-Paquette S., Lévesque S. (2025). Phenotyping the Responses to Systemic Corticosteroids in the Management of Asthma Attacks (PRISMA). Eur. Respir. J..

[84] Celis-Preciado C.A., Leclerc S., Duval M., Cliche D.O., Larivée P., Lemaire-Paquette S., Lévesque S., Côté A., Lachapelle P., Couillard S. (2023). Phenotyping the Responses to Systemic Corticosteroids in the Management of Asthma Attacks (PRISMA): Protocol for an Observational and Translational Pilot Study. BMJ Open Resp. Res..

[85] Ramakrishnan S., Russell R.E.K., Mahmood H.R., Krassowska K., Melhorn J., Mwasuku C., Pavord I.D., Bermejo-Sanchez L., Howell I., Mahdi M. (2025). Treating Eosinophilic Exacerbations of Asthma and COPD with Benralizumab (ABRA): A Double-Blind, Double-Dummy, Active Placebo-Controlled Randomised Trial. Lancet Respir. Med..

[86] Rodrigues H.C., Martins C., Fragoso E., Lopes C., Azevedo P. (2023). Mepolizumab in Severe Asthma Exacerbation in a Respiratory ICU—A Successful Off-Label Use. Pulmonology.

[87] Grasmuk-Siegl E., Xhelili E., Doberer D., Urban M.H., Valipour A. (2024). Tezepelumab in a Case of Severe Asthma Exacerbation and Influenza-Pneumonia on VV-ECMO. Respir. Med. Case Rep..

[88] Rupani H., Busse W.W., Howarth P.H., Bardin P.G., Adcock I.M., Konno S., Jackson D.J. (2024). Therapeutic Relevance of Eosinophilic Inflammation and Airway Viral Interactions in Severe Asthma. Allergy.

